# Evaluating the validity of the simplified conjoint-recognition model

**DOI:** 10.3758/s13423-026-02956-x

**Published:** 2026-07-20

**Authors:** Xinya Liu, C. J. Brainerd

**Affiliations:** Department of Psychology, Cornell University, Martha Van Rensselaer Hall, Ithaca, NY 14853 USA

**Keywords:** Conjoint recognition, Fuzzy-trace theory, Recollection, False memory

## Abstract

The distinction between verbatim and gist traces has been central to various fields of cognitive research, especially false memory, judgment and decision-making, cognitive development, and aging. The conjoint-recognition paradigm was developed to quantify the contributions of verbatim and gist traces to various tasks in those fields. A simplified version of the original paradigm has been devised and has been implemented in several recent studies. However, the validity of this methodology and the memory processes it measures remain uncertain. To address the problem, we first conducted factor analyses at the level of experiment conditions and then a posterior factor analysis to determine whether the simplified paradigm decomposes verbatim and gist traces in the same manner as the full paradigm. Second, we evaluated the sensitivity and specificity of verbatim and gist parameters to a series of experimental manipulations to examine whether the simplified model satisfies convergent and discriminant validity criteria. Two conclusions emerged. On the one hand, the simplified conjoint-recognition paradigm is a reliable and valid method of separating the contributions of verbatim and gist memory in the tasks to which it has been applied. On the other hand, the specific test format increases the difficulty of recollection rejection.

Fuzzy-trace theory (FTT) relies on two memory representations, verbatim and gist traces, to account for a wide range of episodic memory phenomena. Both types of traces can support illusory memories, and both are subject to the influences of several encoding, retrieval, and individual differences variables. The conjoint-recognition paradigm (CR; Brainerd et al., 1999) and an accompanying measurement model were developed to separate the verbatim and gist effects of such variables, and they have been extensively used for that purpose (for a review, see Brainerd et al., 2022). A simplified CR paradigm and model have also been developed (Stahl & Klauer, 2008, 2009), and they have generated a parallel literature on some of the same variables. However, unlike the original paradigm, the validity and reliability of simplified CR have not been closely examined. That was the objective of the present study. To do that, we explicate the methodology of the simplified paradigm and its associated measurement model. Then, we review the key findings that the model has generated to date. First, however, as necessary background, we sketch the original CR technology, along with the theoretical ideas that motivated it.

## Background: Conjoint recognition

### Original CR model

FTT posits that people store two dissociated records of experience in parallel, verbatim traces and gist traces, which are subsequently retrieved in a parallel, dissociated manner (e.g., Brainerd & Reyna, 2001). Verbatim traces capture surface features of items, such as spelling, font, color, and the contexts in which they are embedded. Gist traces capture items’ semantic and relational content. Verbatim and gist traces are opponent processes with respect to false memory. Verbatim traces of encoded items support correct rejection of semantically similar false items (“No, I didn’t read *maple*, I read *pine, oak,* and *willow*.”), whereas gist traces of the same items (“I read the names of a few trees.”) support false acceptance. The two types of traces are also distinguished by their forgetting rates. As time passes, verbatim traces become inaccessible more rapidly than gist traces (Brainerd et al., 1999).

A third distinction between the two types of traces is developmental. Although memory for both the literal form of experience and its semantic content improve during child-to-adult development, gist memory matures more slowly and continues longer (Brainerd et al., 2012a, b). This particular feature leads to the counterintuitive prediction of developmental reversals—that false memories that stem from semantic relations that connect items can be less frequent in children as in comparison with adults. Many examples of these developmental reversals have been reported (for a review, see Brainerd & Reyna, 2023).

The original CR model measures variations in verbatim and gist memory across different conditions and participant groups. The key feature of the CR paradigm is that it enriches the design of standard old–new recognition with two other conditions, with the three conditions being designated V (for verbatim), G (for gist), and VG (for verbatim + gist). When these conditions are factorial crossed with three types of test items (old, new similar, and new novel), the paradigm has 9 degrees of freedom (free empirical probabilities) for modeling. In CR experiments, V is a standard old–new condition in which participants are instructed to accept only old items; G is a complementary condition in which participants are instructed to accept only new-similar items; and VG is a disjunctive condition in which participants are told to accept both old and similar items.

According to FTT, verbatim and gist retrieval generate different response patterns in these conditions. In the original version of the model, for old items, verbatim retrieval yields acceptance under V and VG instruction but rejection under G instruction, whereas gist retrieval yields acceptance in all three conditions. For similar distractors, verbatim retrieval yields acceptance under G and VG instructions but rejection under V instructions, whereas gist retrieval yields acceptance in all three conditions. For novel distracts, verbatim and gist retrieval both yield rejection in all three conditions.

A measurement model was defined over this paradigm that provides estimates of six memory parameters and three bias parameters on a common ratio scale. The process definitions of these parameters appear in Table 1. The parameter set can be thought of as consisting of three verbatim retrieval parameters (*I*,* E*, and *R*), three gist retrieval parameters (*P*,* S*_*t*_, and *S*_*r*_), and three bias parameters (*b*_*v*_,* b*_*g*_, and *b*_*vg*_). The *E* and *P* parameters incorporates some phenomena that were not included in the original CR model. One phenomenon, which is measured by *E*, is that old items can sometimes retrieve verbatim traces of other old items, rather than their own verbatim traces. This produces acceptance for old items in the G and VG conditions but rejection in the V condition. The other phenomenon, which is measured by *P*, is that similar distractors can sometimes retrieve gist traces that are so strong that they foment illusory vivid recollection of their “presentation.” This produces acceptance of similar distractors in the V and VG conditions but rejection in the G condition.

**Table 1:** Parameter definitions for the conjoint-recognition model

Parameter	Definition
Retrieval parameters: True memory	
*I*	Identity: The probability that old items retrieve verbatim traces of their own presentations, which produces vivid recollection of their earlier presentation.
*E*	Erroneous recollection rejection: When identity fails, this is the probability that old items retrieve verbatim traces of the presentations of related old items, which produces vivid recollection of the related old items’ earlier presentation.
*S*_*t*_	Semantic familiarity: When identity and erroneous recollection rejection fail, this is the probability that old items retrieve gist traces shared by themselves and related old items, which produces feelings of semantic familiarity.
Retrieval parameters: False memory	
*R*	Recollection rejection: The probability that new-similar items retrieve verbatim traces of related old items, which produces vivid recollection of the related old items’ presentation.
*P*	Phantom recollection: When recollection rejection fails, this is the probability that new-similar items retrieve stronger gist traces of related old items, which produces illusory recollection of the presentation of new-similar items.
*S*_*r*_	Semantic familiarity: When recollection rejection and phantom recollection fail, this is the probability that new-similar items retrieve weaker gist traces of related old items, which produces feelings of semantic familiarity.
Bias parameters	
*b*_*v*_	This is the one-high-threshold bias probability for V instructions.
*b*_*g*_	This is the one-high-threshold bias probability for G instructions.
*b*_*vg*_	This is the one-high-threshold bias probability for VG instructions.

To obtain estimates of the parameters in Table 1, the free empirical probabilities are expressed as functions of these parameters. The relevant expressions are as follows:

1 $$p\mathrm{(A)}T\_V\text{ = }I\text{ + (1 - }I\text{)(1 }- \, E\mathrm{)}St\text{ + (1 }- \, I\text{)(1 }- \, E\text{)(1 }- \, St\mathrm{)}bv,$$

2 $$p\mathrm{(A)}T\_G\text{ = (1 }- \, I\mathrm{)}E\text{ + (1 }- \, I\text{)(1 }- \, E\mathrm{)}St\text{ + (1 }- \, I\text{)(1 }- \, E\text{)(1 }- \, St\mathrm{)}bg\mathrm{,}$$

3 $$p\mathrm{(A)}T\_VG\text{ = }I\text{ + (1 }- \, I\mathrm{)}E + \text{(1 }- \, I\text{)(1 }- \, E\mathrm{)}St\text{ + (1 }- \, I\text{)(1 }- \, E\text{)(1 }- \, St\mathrm{)}bvg\mathrm{,}$$

4 $$p\mathrm{(A)}RD\_V\text{ = (1 }- \, R\mathrm{)}P\text{ + (1 }- \, R\text{)(1 }- \, P\mathrm{)}Sr \text{+ (1 }- \, R\text{)(1 }- \, P\text{)(1 }- \, Sr\mathrm{)}bv\mathrm{,}$$

5 $$p\mathrm{(A)}RD\_G\text{ = }R\text{ + (1 }- \, R\text{)(1 }- \, P\mathrm{)}Sr\text{ + (1 }- \, R\text{)(1 }- \, P\text{)(1 }- \, Sr\mathrm{)}bg\mathrm{,}$$

6 $$p\mathrm{(A)}RD\_VG\text{ = }R\text{ + (1 }- \, R\mathrm{)}P\text{+ (1 }- \, R\text{)(1 }- \, P\mathrm{)}Sr\text{ + (1 }- \, R\text{)(1 }- \, P\text{)(1 }- \, Sr\mathrm{)}bvg\mathrm{,}$$

7 $$p\mathrm{(A)}UD\_V\text{ = }bv\mathrm{,}$$

8 $$p\mathrm{(A)}UD\_G\text{ = }bg\mathrm{,}$$

9 $$p\mathrm{(A)}UD\_VG\text{ = }bvg\mathrm{,}$$

where the *p*(A)*ij* are acceptance probabilities that run over the three item types [*i* = old (T), similar (RD), and novel (UD)] and the three instructional conditions (*j* = V, G, and VG). The model is fit to data by imposing some equality constraint that reduces the number of theoretical parameters from nine to eight. Although multiple constraints have been used (e.g., *E* = *P*, *S*_*t*_ = *S*_*r*_), the traditional constraint is *b*_*g*_ = *b*_*vg*_.

The verbatim parameters (*I*,* E*, and *R*) supply independent measurements of three types of verbatim retrieval: when old items retrieve their own verbatim traces, when old items retrieve verbatim traces of *other* old items, and when similar items retrieve verbatim traces of old items. Second, the gist parameters (*P*,* S*_*t*_, and *S*_*r*_) supply independent measurements of three types of gist retrieval: when similar distractors retrieve gist traces of old items that are so strong that they induce phantom recollection of similar distractors, when old items retrieve their own gist traces, and when similar distractors retrieve gist traces of old items that do not produce phantom recollection. Third, the three bias parameters (*b*_*v*_,* b*_*g*_, and *b*_*vg*_) supply independent measurements of the tendency to accept novel distractors under three types of instructions.

In the original CR paradigm, the instructions factor (V, G, and VG) is manipulated between participants. Hence, as a practical manner, the number of participants per experiment that is required to achieve acceptable levels of statistical power could be substantially reduced if instructions were manipulated within participants (Stahl & Klauer, 2008). Stahl and Klauer (2008, 2009) provided such a repeated-measures procedure, the simplified CR paradigm, which altered both the methodology of the original paradigm and the structure of its measurement model.

These nine variables are the identifiable parameters of the multinomial version of the conjoint-recognition model.

### Simplified CR model

The simplified paradigm also aims to separate the effects of verbatim and gist retrieval on true and false memory. The major modification is a multiple-choice testing format, in which participants identify test probes (old, similar distractors, and novel distractors) as old, similar or novel. Participants are informed of the three types of probes that will be presented during the test phase. They are required to choose the “old” option if they believe the current test probe is old, the “similar” option if they believe the current test probe is a similar distractor, and the “novel” option if they believe the test probe is a novel distractor. This multiple-choice procedure reduces the number of instructional conditions from three to one and reduces participant sample sizes. However, it also reduces the number of free empirical probabilities from nine to six because there are only 2 degrees of freedom in the three choice options for each test probe.

A measurement model was defined over this procedure that contains verbatim and gist retrieval parameters for old items (*V*_*t*_ and *G*_*t*_) and for similar distractors (*V*_*r*_ and* G*_*r*_). It also contains two bias parameters (*a* and *b*). Old items can retrieve their verbatim traces (with probability *V*_*t*_) and be judged as old. Similar distractors can retrieve verbatim traces of related old items (with probability *V*_*r*_) and be judged to be similar distractors. If verbatim traces are inaccessible, test probes can retrieve gist traces of old items (with probability *G*_*t*_ or* G*_*r*_). In this case, the test item is judged to be old with probability *a*, or it is judged to be similar with probability 1-*a*. When gist traces are not accessible (with probability 1-*G*_*t*_ or *1-G*_*r*_), the test item is judged to be old or similar with probability *b* or it is judged to be novel with probability 1-*b*. For probes that are guessed to be either old or similar, they are identified as old with a probability of *a* and as similar with probability 1-*a*. Thus, whereas *b* is a conventional one-height-threshold bias parameter like those in the original CR model, *a* is not.

The model’s expressions appear in the following six equations, where the items in the parentheses, T, RD, UD, denote judgments made by subjects being old, similar, and novel respectively, and the subscripts T, RD and UD, denote old, similar, and novel items, respectively. For example, *p*(RD)_T_ refers to the probability of judging an old item to be new but similar.

10 $$p\mathrm{(T)}T\text{ = }Vt\text{ + (1} - Vt\mathrm{)}Gta\text{ + (1} - Vt\mathrm{)(1} - \, Gt\mathrm{)}ba\mathrm{,}$$

11 $$p\mathrm{(RD)}T\text{ = (1} - Vt\mathrm{)}Gt\mathrm{(1} - \, a\text{) + (1} - Vt\mathrm{)(1} - \, Gt\mathrm{)}b\mathrm{(1} - \, a\mathrm{),}$$

12 $$p\mathrm{(UD)}T\text{ = (1} - Vt\mathrm{)(1} - \, Gt\mathrm{)(1} - \, b\mathrm{),}$$

13 $$p\mathrm{(T)}RD\text{ = (1} - Vr\mathrm{)}Gra\text{ + (1} - Vr\mathrm{)(1} - \, Gr\mathrm{)}ba\mathrm{,}$$

14 $$p\mathrm{(RD)}RD\text{ = }Vr\text{ + (1} - Vr\mathrm{)}Gr\mathrm{(1} - \, a\text{) + (1}- Vr\mathrm{)(1} -Gr)b\mathrm{(1} - \, a\mathrm{),}$$

15 $$p\mathrm{(UD)}RD\text{ = (1} - Vr\mathrm{)(1} - \, Gr)\mathrm{(1} - \, b\mathrm{),}$$

16 $$p\mathrm{(T)}UD\text{ = }ba\mathrm{,}$$

17 $$p\mathrm{(RD)}UD\text{ = }b\text{(1 }- \, a\mathrm{),}$$

18 $$p\mathrm{(UD)}UD = 1- \, b,$$

As the model has six parameters and a data space with six free probabilities, it leaves no degrees of freedom for model fitting. Hence, an identifying restriction of some sort (e.g., *V*_*r*_ = 0) is needed to test model fit. Alternatively, a hierarchical Bayesian model, which is able to estimate group-level and individual-level parameters, can generate goodness-of-fit comparisons between observed and predicted frequencies (e.g., see Greene & Naveh-Benjamin, 2020). Similar to the original CR paradigm, the simplified version has been used in several studies to parse the contributions of verbatim and gist memory to the effects of some classic episodic memory variables (e.g., age, divided attention, encoding time, repetition; see Abadie & Camos, 2019; Greene & Naveh-Benjamin, 2020, 2022b, 2023a, b, c; Nieznański & Obidziński, 2019; Nieznański & Tkaczyk, 2017; Nieznański et al., 2024).

Some initial parameter validation data were provided by Stahl and Klauer (2008, 2009), who examined the convergent and discriminant validity of the parameters. The former refers to parameters’ sensitivity to experimental manipulations that are known to affect the corresponding retrieval processes, whereas the latter refers to parameters’ insensitivity to manipulations that should not affect the corresponding retrieval processes (Klauer et al., 2011). Manipulations that targeted *V*_*t*_, *G*_*t*_, *V*_*r*_, and* G*_*r*_ were investigated. The first verbatim manipulation was repeated presentation of list items, which had a reliable effect on one verbatim parameter (*V*_*t*_) but did not affect either gist parameter (*G*_*t*_ or* G*_*r*_). The second verbatim manipulation, which only affected *V*_*r*_, was to test similar distractors immediately after testing their corresponding old items (e.g., testing the similar distractor *couch* immediately after testing the old item *sofa*). Finally, both gist parameters were increased by a gist manipulation that involved category size. Specifically, with categorized lists, both gist parameters increased as the number of exemplars per category increased.

### The present study

The large data base on the original CR paradigm has established that the model in Table 1 delivers acceptable fits to data that were generated by a wide range of materials, experimental conditions, and participant groups, including groups with neurocognitive disease (Brainerd & Reyna, 2018). The factor structure of the model’s parameter space has been identified, and the parameters’ convergent and discriminant validity has been established (for a review, see Brainerd et al., 2022). What about the simplified paradigm? The factor structure of its parameter space is currently unknown, especially whether the structure conforms to theoretical predictions about it. Also, beyond Stahl and Klauer’s (2008, 2009) initial data, the models’ convergent and discriminant validity have not been examined. Obviously, both questions need to be answered before we can say whether the verbatim and gist retrieval processes that are measured by the simplified model are comparable to those that are measured by the original model.

We investigated these questions with the accumulated database on the simplified model. We collected relevant articles through Web of Science searches using key words and also searching for articles that had cited certain key references. We then selected all those that had implemented the simplified CR procedure and its measurement model. A total of 26 articles were identified, which include 176 conditions, all of which were included in our analyses.

We extracted three types of results from these articles. First, we considered the baseline issue of fit; that is, whether the simplified model, like the original model, usually delivers acceptable fits to the data space over which it is defined. Second, we conducted an exploratory factor analysis of the simplified model’s parameter space, to determine whether it conforms to theoretical expectations and whether it matches the original model’s factor structure. In that connection, FTT specifies that verbatim and gist parameters should load on distinct orthogonal factors because they measure dissociated retrieval processes that operate in parallel. That is the pattern exhibited by the original model (Brainerd et al., 2014, 2022). Third, we considered whether the simplified model’s verbatim and gist parameters display sensitivity and specificity with respect to experimental manipulations. More explicitly, do manipulations that target verbatim memory affect the verbatim parameters (sensitivity) but not the gist parameters (specificity), and conversely for manipulations that target gist memory?

The analyses were conducted with the *MPTinR* package (Singmann & Kellen, 2013), the *psych* package (Version 2.3.3; Revelle, 2023) and the *TreeBUGS* package (Heck et al., 2018) in R (Version 4.3.0; R Core Team, 2023). All data and analysis scripts are available at https://osf.io/f739y/overview?view_only=595a8885df9a48c8ba73464b88538f54

## Results

### Fit of the simplified model

All the studies reported statistically acceptable fits to experimental data. However, relative to Eqs. 12–18, the fit results in some articles were pursuant to certain model modifications. Those modifications were of three sorts: adding parameters, restricting the values of certain parameters, and adopting alternative statistical methods. Examples of the first modification include (a) introducing a different guessing parameter, *a*_*b*_, to distinguish between guessing “old” when gist memory is available and guessing “old” when gist memory is not available; (b) introducing a phantom recollection parameter, *P*, to capture illusory vivid phenomenology (e.g., Stahl & Klauer, 2009); and (c) introducing a fuzzy judgment parameter, *F*, that measures memory for distractors that vary in resemblance to old items. Each of these changes constitutes a different parameterization than the core parameterization in Eqs. 12–18. Turning to the second modification, restrictions to parameters consist of setting some parameters to zero (e.g., *V*_*r*_), and imposing equivalence relations among different parameters to achieve identifiability. The third modification is to adopt a hierarchical Bayesian version of the model to accommodate individual differences. Studies that incorporated these modifications are summarized in Table 2.

**Table 2:** Summary of modifications

Author	Statistical method	Model parameters
Abadie et al. 2017	Hierarchical Bayesian latent-trait	Six parameters
Abadie et al. 2021	Hierarchical Bayesian latent-trait	Six parameters
Abadie and Rousselle, 2023	MPT	Six parameters + *P*
Abadie and Guette 2025	Hierarchical Bayesian latent-trait	Six parameters
Gong et al. 2016	MPT	Six parameters + *P* + *a*_*b*_
Greene and Naveh-Benjamin, 2020	Hierarchical Bayesian latent-trait	Six parameters + *F* + *a*_*b*_
Greene and Naveh-Benjamin, 2022a	Hierarchical Bayesian latent-trait	Six parameters + *a*_*b*_
Greene and Naveh-Benjamin, 2022b	Hierarchical Bayesian latent-trait	Six parameters + *a*_*b*_
Greene and Naveh-Benjamin, 2022c	Hierarchical Bayesian latent-trait	Six parameters + *F* + *a*_*b*_
Greene and Naveh-Benjamin, 2023a	Hierarchical Bayesian latent-trait	Six parameters + *F* + *a*_*b*_
Greene and Naveh-Benjamin, 2023b	Hierarchical Bayesian latent-trait	Six parameters + *F* + *a*_*b*_
Greene and Naveh-Benjamin, 2023c	Hierarchical Bayesian latent-trait	Six parameters + *F* + *a*_*b*_
Greene and Naveh-Benjamin, 2024	Hierarchical Bayesian latent-trait	Six parameters + *a*_*b*_
Greene et al. 2024	Hierarchical Bayesian latent-trait	Six parameters + *a*_*b*_
Macera and Daurat, 2018	MPT	Six parameters + *P*
Nieznański and Tkaczyk, 2017	MPT	Six parameters + *P*
Nieznański and Obidziński, 2019	MPT	Six parameters + *P*
Nieznański et al. 2024	MPT	Six parameters + *P*
Obidziński and Nieznański, 2017	MPT	Six parameters + *P*
Stahl and Klauer, 2008	MPT	Six parameters with *a* and *b* equal across experiment manipulations
Stahl & Klauer, 2009	MPT	Six parameters with *a* and *b* equal across experiment manipulations + *P*_*r*_

MPT = multinomial processing tree model. Six parameters = *V*_*t*_, *V*_*r*_, *G*_*t*_, *G*_*r*_, *a*, and *b*. *P* = phantom recollection of related distractors. *F* = fuzzy judgment of unrelated distractors. *a*_*b*_ = guessing process conditioned on guessing process *b*

**Fig. 1:**
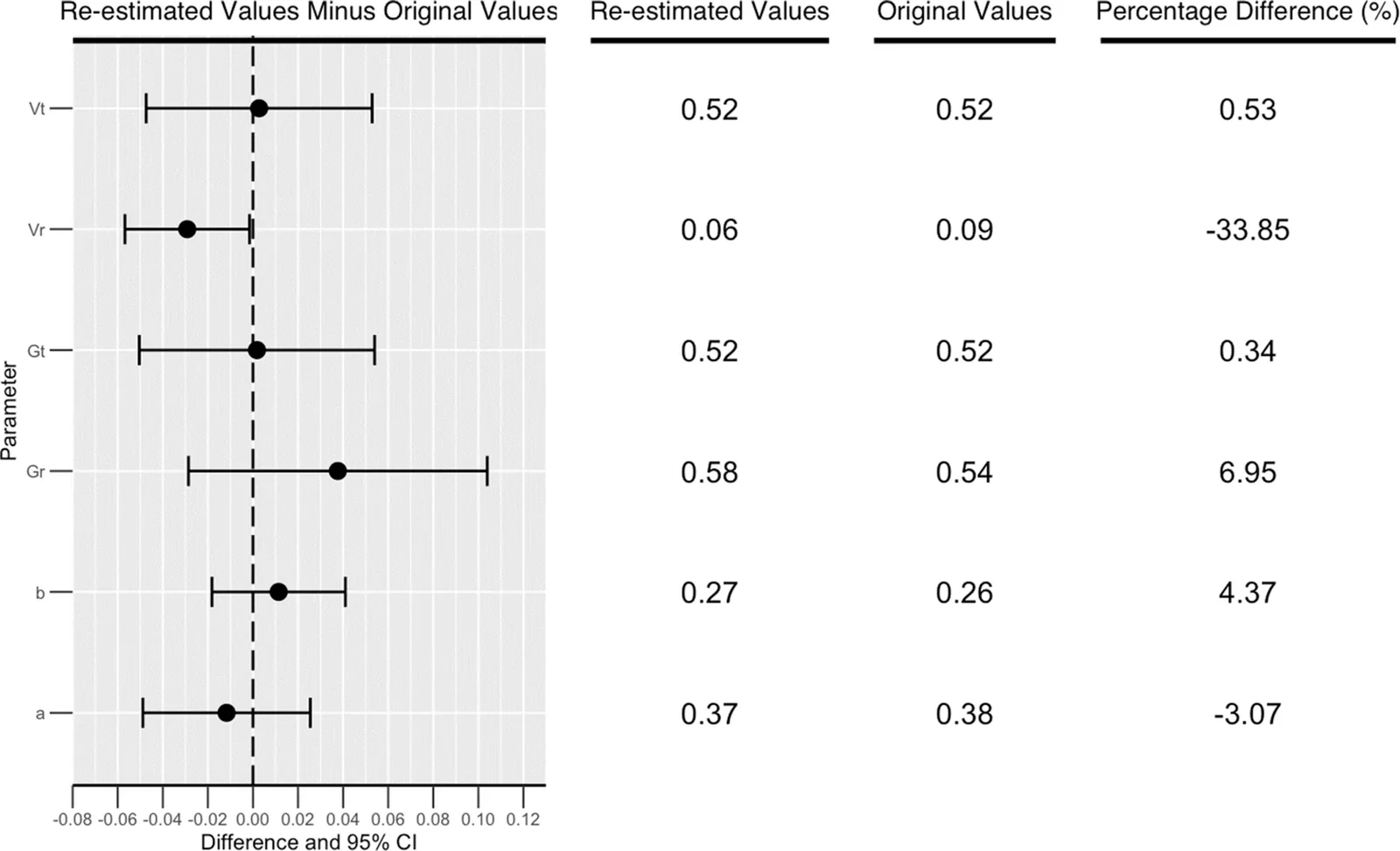
Forest plot for re-estimated values versus original values. *Note.* Percentage Difference = (Re-estimated Value − Original Value) / Original Value

The first two types of modifications pose an obstacle to a review such as this, whose objective is to isolate coherent patterns of parameter behavior across different studies. The obstacle is that it has long been known (Brainerd et al., 1982) that estimates of the same parameter (say, *G*_*t*_) in different studies cannot be compared if (a) they were computed under different parameterization of a model, or (b) some were computed with hierarchical versions of a model and others were computed with non-hierarchical versions. To remove this problem, we obtained the raw data of the studies that we reviewed, and we re-fit all the data with a single parameterization of the model (Eqs. 10–18) and a single method of estimation. The analyses were conducted with the *MPTinR* package in R (Singmann & Kellen, 2013). The mean values of the re-estimated parameters, across all experiments, along with the means of their original published values, appear in Fig. 1. The bottom-line is that the discrepancies between the two sets of estimates were modest. The largest absolute discrepancy was in *G*_*r*_ (.04) and the largest percentage change was in *V*_*r*_ (34%).

### Factor analysis at the condition level

We conducted an exploratory factor analysis to determine whether the parameters of the simplified model generate the type of factor space that theory anticipates. The analyses were conducted with the psych package in R (Revelle, 2023). The data inputs were the estimates of the model’s six parameters for all the conditions of the experiments that were generated by our reanalysis of the data. As noted, FTT expects that verbatim parameters will load together on the same factor, and gist parameters will load together on a different orthogonal factor. As also noted, that pattern has been observed for the original CR model. This can be seen in Table 3, which displays the rotated factor loadings for 478 data sets that were reviewed by Brainerd et al. (2022). As can be seen, there is a verbatim factor (Factor 3) on which the *I* and *R* parameters load strongly, a gist factor (Factor 1) on which the *S*_*t*_ and *S*_*r*_ parameters load strongly, and a third factor (Factor 2), on which the *E* and *P* parameters load strongly. In line with various experimental results reviewed by Brainerd et al. (2022), Factor 3 is interpreted as measuring retrieval of verbatim traces of old items’ presentations, Factor 1 is interpreted as measuring retrieval of gist traces of old items’ semantic content, and Factor 2 is interpreted as measuring phantom recollection of similar distractors.

**Table 3:** Rotated factor loadings of the full CR model in Brainerd et al. (2022)

Parameters	Factor 1 Gist retrieval	Factor 2 Phantom recollection	Factor 3 Verbatim retrieval
*I*			0.61
*E*		0.92	
*S*_*t*_	0.87		
*R*			0.92
*P*		0.85	
*S*_*r*_	0.85		

Turning to the simplified CR model, the theoretical expectation is that its factor space will consist of at least two orthogonal dimensions, one on which the two verbatim parameters load and one on which the two gist parameters load, with the two bias parameters loading on the gist dimension because they are conditioned on the presence or absence of gist memory (see Brainerd et al., 2022). An exploratory factor analysis^1^ with orthogonal rotation revealed a three-dimensional space, which is displayed in Table 4. The three factors accounted for 76.8% of total variance. Parameter loadings and factor scores appear in Panels A and B, respectively. As shown in Table 4, the two gist parameters loaded together on the same factor along with the *b* bias parameter, consistent with theory. However, the two verbatim parameters did not load on the same factor. There was a verbatim factor on which only *V*_*r*_ loaded. Then, there was another verbatim factor, which displayed a nearly perfect loading for *a* and on which *V*_*t*_ also loaded negatively. We ran a forced two-factor model to explore *V*_*r*_’s relation with *V*_*t*_. As illustrated in Panel A of Table 5, *V*_*t*_ and *V*_*r*_ loaded on the same factor with *a*. However, this model delivered an unacceptable fit to the data. We next removed *V*_*r*_ and conducted an exploratory factor analysis of the remaining five parameters. The results appear in Panel B of Table 5, with 65.5% of the variance accounted for. The observed patterns suggest that excluding *V*_*r*_ did not affect parameter structure: verbatim and gist processes were again dissociated.

**Table 4:** Results of factor analysis on the full dataset

Panel A. Rotated factor loading			
	Factor 1	Factor 2	Factor 3
*V*_*t*_		−0.73	
*V*_*r*_			0.99
*G*_*t*_	0.84		
*G*_*r*_	0.83		
*a*		1.00	
*b*	−0.64		
Panel B. Mean factor scores			
	Factor 1	Factor 2	Factor 3
*V*_*t*_	−0.01	−0.01	−0.01
*V*_*r*_	0.07	−0.08	1.00
*G*_*t*_	0.42	0.01	−0.01
*G*_*r*_	0.48	−0.04	0.00
*a*	−0.05	0.99	0.11
*b*	−0.18	−0.01	0.00

The factor loadings in Panel A followed the usual convention (see Brainerd et al., 2008) of regarding loadings <.40 as unreliable

**Table 5:** Results of factor analysis based on alternative model specifications

Panel A			Panel B			Panel C		
	Factor 1	Factor 2		Factor 1	Factor 2		Factor 1	Factor 2
*V*_*t*_		1.01	*V*_*t*_		−0.95	*V*_*t*_		0.43
*V*_*r*_		0.32	*G*_*t*_	0.88		*V*_*r*_		0.80
*G*_*t*_	0.87		*G*_*r*_	0.80		*G*_*t*_	0.83	
*G*_*r*_	0.80		*a*		0.74	*G*_*r*_	0.84	
*a*		−0.65	*b*	−0.61		*B*	−0.66	−0.30
*b*	−0.62							

Panel A shows results of the forced two-factor analysis. Panel B shows results derived from sub data excluding *V*_*r*_. Panel C shows results derived from sub data excluding *a*. Factor loadings <.30 as unreliable. Rotation = varimax

A possible explanation for the fact that *V*_*r*_ loaded on a separate factor is that the correlation between *V*_*t*_ and *a* was so strong (−0.71) that the two parameters were assigned to the same factor while *V*_*r*_ had only a moderate positive correlation with *V*_*t*_ (.34) and almost no correlation with *a* (−0.05) and, hence, drifted to another factor (see Table 6). This hypothesis was corroborated by a parallel factor analysis removing values of *a*, as reported in Panel C of Table 5. Without *a*, *V*_*t*_ loaded with *V*_*r*_ on one factor, and the *G*_*t*_, *G*_*r*_ and *b* loaded together on the other factor, with *b* showing a minor cross-loading on the verbatim factor. This model accounted for 56.6% of total variance.

**Table 6:** Correlation matrix of parameter values at condition level

	*V*_*t*_	*V*_*r*_	*G*_*t*_	*G*_*r*_	*a*	*B*
*V*_*t*_	1					
*V*_*r*_	0.34	1				
*G*_*t*_	−0.08	−0.01	1			
*G*_*r*_	−0.14	−0.22	0.71	1		
*a*	−0.71	−0.05	0.09	0.12	1	
*b*	−0.12	−0.25	−0.53	−0.47	0.08	1

Taken together, these results support three conclusions about the simplified CR model. First, consistent with theory, the verbatim and gist parameters measure different aspects of retrieval because they did not load on a common factor. Second, also consistent with theory, the two gist parameters appear to measure the same aspect of memory because they loaded strongly on the same factor. Third, the relation between *V*_*t*_ and *V*_*r*_ was less clear as they loaded on separate factors. This was reconciled after removing *a* from the analysis, suggesting that the correlation between *V*_*t*_ and *V*_*r*_ was obscured by the strong association between *V*_*t*_ and *a*. Two explanations are available. Frist, the simplified CR test makes recollection rejection especially difficult, leading to relatively low variability in *V*_*r*_ and weak association of *V*_*t*_ and *V*_*r*_. Second, the current analysis covered a limited number of studies which were skew toward lower values (especially zero) of *V*_*r*_. The first explanation points to the inherent ability of the simplified CR paradigm to capture recollection rejection, which we will explore in more detail in the discussion section.

The second explanation cannot be directly evaluated with extant data but can be examined from another angle: When experiments produce nonzero values of *V*_*r*_, what does the structure of the parameter space look like? A simple approach is to remove the zero values of *V*_*r*_ and run a parallel factor analysis on the sub corpus with *V*_*r*_ > 0. However, this method failed to account for sufficient variance, RMSEA = 0.26, 95% credible interval [0.18, 0.35]. Another approach is to select an experiment that reported nonzero *V*_*r*_ values and examine parameter structure at the level of individual participants, which will be discussed in the next section. Regardless, it should be noted that low values of *V*_*r*_ and their modest correlations with *V*_*t*_ are important points of difference between the databases of the original and simplified CR models. In the database for the original model, the corresponding verbatim parameters (*I* and *R*) are strongly correlated, and the mean value of the recollection rejection parameters is substantially larger.

### Posterior factor analysis

The parameter values that were used in the previous section were derived from aggregation across all participants in each condition of individual experiments. Many experimental conditions failed to elicit reliable recollection rejection, for example, conditions with long retention interval, long word lists, or elderly participants. Consequently, *V*_*r*_ in these conditions remained close to zero and did not covary with *V*_*t*_ as much as *a* did. Further, available studies may contain a disproportionate concentration of low values for *V*_*r*_, which would invalidate the factor analysis results, if true. In the last section, conditional-level factor analysis on a sub corpus with *V*_*r*_ > 0 did not produce reliable fit. Therefore, in this section, we adopt a posterior factor analysis approach to investigate parameter structure for two studies that reported nonzero estimates of *V*_*r*_.

We used the latent-trait MPT model adapted from Greene et al. (2024) for the posterior factor analysis. Latent-trait MPT model assumes that individuals adhere to a common processing tree but differ on a latent distribution, supplying a group-level estimation of parameters and also an individual-level estimation (Klauer, 2010). Latent-trait MPT model is a hierarchical Bayesian approach which relies on Bayesian inference with Markov-chain Monte Carlo (MCMC) sampling. In each MCMC draw, the model generates group-level parameter estimates, individual-level parameter estimates and a group-level correlation matrix of parameters. The posterior correlation matrices in the MCMC draws thus enable the posterior factor analysis. We examined the parameter structure for each MCMC iteration and computed posterior probabilities that verbatim or gist parameters loaded on the same factor. Our analysis involved the following steps:

- Fitting a latent-trait MPT model to the data of individual participants with *TreeBUGS* package (Heck et al., 2018) in R;
- Determining the number of factors for all the correlation matrices generated by the program;
- Conducting factor analyses of the matrices;
- Extracting the dominant factor for each parameter—namely, the factor on which the parameter had the highest loading; and
- Comparing dominant factors for each parameter. Parameters are considered to load on the same factor if they have the same dominant factor.

Two papers, Nieznański and Obidziński (2019) and Greene et al. (2024), were selected for this analysis using the following criteria:

- The experiments generated *V*_*r*_ that were significantly >0 (group-level estimates appear in Table 7) and
- The experiments supplied large numbers of Participants × Items replications.

**Table 7:** Group-level parameter estimates in Greene et al. (2024) and Nieznański and Obidziński (2019)

Panel A				Panel B	
Parameter	Estimates			Parameter	Estimates
	Experiment 1	Experiment 2	Experiment 3		
*V*_*t*_*_small*	0.26	0.25	0.3	*V*_*t*_	0.58
*V*_*t*_*_large*	0.15	0.26	0.09	*V*_*r*_	0.03
*V*_*r*_	0.09	0.04	0.06	*G*_*t*_	0.41
*G*_*t*_*_ small*	0.59	0.44	0.33	*G*_*r*_	0.30
*G*_*t*_*_ large*	0.36	0.29	0.3	*a*	0.41
*G*_*r*_*_ small*	0.44	0.18	0.17	*b*	0.32
*G*_*r*_*_ large*	0.24	0.18	0.14		
*a*	0.68	0.73	0.63		
*b*	0.39	0.5	0.48		
*a*_*b*_	0.24	0.31	0.27		

Panel A represents parameter estimates derived from Greene et al. (2024). Panel B represents parameter estimates derived from Nieznański and Obidziński (2019)

For instance, Nieznański and Obidziński (2019) administered a memory test with 48 old words, 48 new words that were orthographically related to the old words, and 48 new and unrelated words. A total of 146 subjects participated in the experiment.

In Greene et al. (2024), participants studied categorized images in small blocks (two images per block) and large blocks (six images per block), performed a classic old-new recognition test immediately after each block, and then received a long-term memory test in the simplified CR format. On the final test, 72 old images and 72 new-similar images were presented (36 old images per condition and 36 new-similar images per condition), together with 72 new unrelated images. We combined individual response data from the three experiments in the paper, which differed only in the size of the images and presentation format (sequential display in Experiment 1a and 1b vs. simultaneous display in Experiment 2), yielding a total sample of 120 participants.

## Results: Nieznański and Obidziński (2019)

We fitted the simplified CR model as defined in Eqs. 10-18 to data aggregated across participants. We fitted a latent-trait MPT model with the same parameterization to individual response data and used the procedures described above to investigate the structure of the parameter space. The hierarchical model produced 16,500 MCMC samples along with parameter correlation matrices. Next, we computed eigenvalues of each matrix to determine the number of latent factors, and 74.38% of all the 16,500 matrices supported a three-factor model, 25.50% produced a two-factor model, and 12.12% supported a four-factor model. Therefore, we fitted a three-factor model without rotation^2^ to each matrix. Model fit was overall satisfactory, mean RMSEA = 0.020, 95% credible interval [5.15×10^−7^, 6.49×10^−2^]. Note that although no rotation was applied, orientations across each matrix were not necessarily the same. Consequently, it is invalid to compare factor loadings across samples. To investigate parameter clustering, we operationalized the dominant factor of a parameter in a matrix as the factor on which the parameter had the largest loading. Having the same dominant factor means that parameters clustered on the same factor. In addition, the mean loadings and 95% credible intervals were computed to determine whether the clustering pattern was reliable.

Results revealed a verbatim clustering: *V*_*t*_ and *V*_*r*_ loaded on the same factor in 66.2% of the samples, with factor loadings above 0.70 and credibly different from zero, as reported in Panel A of Table 8. In other words, the posterior probability that verbatim parameters loaded on the same factor was 66.2%. The two gist parameters loaded on the same factor in only 39.9% of the total sample (see Panel B of Table 8). Further, there was compelling evidence of independence between verbatim and gist processes. Only 11.6% of the samples produced a shared dominant factor for *V*_*t*_, *V*_*r*_, *G*_*t*_, and *G*_*r*_ (see Panel C of Table 8).

**Table 8:** Results of factor analysis on Nieznański and Obidziński (2019)

Panel A			
PostPr	Parameter	Factor loading	95% credible interval
66.2%	*V*_*t*_	**0.77**	[0.52, 1.00]
	*V*_*r*_	**0.73**	[0.44, 1.00]
Panel B			
PostPr	Parameter	Factor loading	95% credible interval
39.9%	*G*_*t*_	**0.62**	[0.30, 0.94]
	*G*_*r*_	**0.79**	[0.51, 1.00]
Panel C			
PostPr	Parameter	Factor loading	95% credible interval
11.6%	*V*_*t*_	**074**	[0.52, 0.95]
	*V*_*r*_	**0.71**	[0.44, 0.94]
	*G*_*t*_	**0.56**	[0.29, 0.85]
	*G*_*r*_	**0.84**	[0.59, 1.00]
Panel D			
PostPr	Parameter	Factor loading	95% credible interval
2.6%	*V*_*t*_	**0.66**	[0.45, 0.85]
	*V*_*r*_	**0.70**	[0.44, 1.00]
	*a*	**0.39**	[−0.63, 0.79]
Panel E			
PostPr	Parameter	Factor loading	95% credible interval
4.7%	*G*_*t*_	**0.60**	[0.32, 0.89]
	*G*_*r*_	**0.68**	[0.47, 0.88]
	*b*	0.51	[−0.28, 0.83]

PostPr = posterior probability that the parameters loaded on the same dominant factor. 95% credible intervals conditional on the specific clustering are presented in brackets. No rotation applied. Loadings were shown in boldface whose 95% credible intervals did not include zero

Next, we assessed whether the loadings of guessing parameters that were identified at the condition level were preserved in the posterior analysis. Only a small proportion of the samples (2.6%) yielded solutions in which the verbatim parameters loaded with the guessing parameter *a*, and the proportion that the gist parameters loaded with the guessing parameter *b* was also very small (4.7%) (see Panel D and E of Table 8).

To sum up, posterior factor analyses on Nieznański and Obidziński’s data (2019) revealed a clear verbatim clustering but an unreliable gist clustering. Also, verbatim and gist parameters were independent, as they did not load on the same dominant factor in the majority of the samples. Further, the guessing parameters did not load consistently with the retrieval parameters, as opposed to the pattern indicated by the condition-level results. We did not detect a strong association between the gist parameters with the current dataset, which could be due to the fact that the type of gist that was measured was not semantic. Here, it must be borne in mind that several false memory experiments have revealed that phonological gist is processed differently than semantic gist (for a review, see Chang & Brainerd, 2021).

### Results: Greene et al. (2024)

Greene et al. (2024) manipulated the size of study blocks within participants. To ensure model fit, we followed the authors’ procedure, allowing *V*_*t*_, *G*_*t*_, and *G*_*r*_ to vary between the large and small block conditions while *V*_*r*_, *a*, *a*_*b*_ and *b* remained constant between the two conditions. Note that an additional guessing parameter *a*_*b*_ was specified to capture guessing when no verbatim or gist memory was retrieved. The guessing parameter *b* was activated, as compared with the guessing parameter *a*, which measured guessing when no verbatim memory was retrieved but gist memory was retrieved. Among all the 16,500 correlation matrices (10 × 10), 53.1% of them produced a three-factor solution, 46.5% produced a two-factor solution, and 0.4% produced a four-factor solution. Therefore, we fitted a three-factor model without rotation to each correlation matrix. This model fitted each matrix well, with mean RMSEA = 0.041, 95% credible interval [0.021, 0.069].

The results revealed a pronounced gist clustering: gist parameters, namely, *G*_*t*__*large*, *G*_*t*_*_small*, *G*_*r*__*large* and *G*_*r*_*_small*, loaded on the same dominant factor in 82.3% of the samples with positive primary loadings. This is shown in Panel A of Table 9. On the other hand, only 37.7% of the samples yielded verbatim clustering. That is, *V*_*t*__*large*, *V*_*t*_*_small* and *V*_*r*_ were grouped on the same factor in roughly one-third of the samples, and the 95% credible intervals of *V*_*t*_*_small* and *V*_*r*_ contained zero cases. Moreover, *V*_*r*_ had a strong tendency to cluster with gist parameters (see Panel C of Table 9), as 69.3% of the samples yielded solutions in which *V*_*r*_, *G*_*t*__*large*, *G*_*t*_*_small*, *G*_*r*__*large* and *G*_*r*_*_small* loaded jointly on one factor, with factor loadings above 0.80 and 95% credible intervals excluding zero. By comparison, only 36.7% of the samples generated solutions in which *V*_*t*__*large*, *V*_*t*_*_small* and the gist parameters loaded on the same factor, which appears in Panel D of Table 9. Finally, contrary to the condition-level results, no consistent pattern was observed for guessing parameters. First, 12% of the sample yielded solutions in which *a*, *V*_*t*_*_small* and *V*_*t*__*large* loaded on the same factor and the factor loadings were not credibly different from zero (see Panel E of Table 9). Moreover, *a*_*b*_ and *b* did not have a marked correlation with gist parameters, as they loaded on the same dominant factor in only 0.3% of the total samples.

**Table 9:** Results of factor analysis on Greene et al. (2024)

Panel A			
PostPr	Parameter	Factor loading	95% Credible Interval
82.3%	*G*_*t*_*_ small*	**0.90**	[0.73, 1.00]
	*G*_*t*_*_ large*	**0.86**	[0.66, 0.99]
	*G*_*r*_*_ small*	**0.90**	[0.72, 0.99]
	*G*_*r*_*_ large*	**0.82**	[0.60, 0.99]
Panel B			
PostPr	Parameter	Factor Loading	95% Credible Interval
37.7%	*V*_*t*_*_small*	−0.39	[−0.99, 0.92]
	*V*_*t*_*_large*	0.70	[−0.41, 0.99]
	*V*_*r*_	**0.78**	[0.53, 0.99]
Panel C			
PostPr	Parameter	Factor Loading	95% Credible Interval
69.3%	*G*_*t*_*_ small*	**0.91**	[0.76, 1.00]
	*G*_*t*_*_ large*	**0.87**	[0.66, 0.99]
	*G*_*r*_*_ small*	**0.91**	[0.73, 0.99]
	*G*_*r*_*_ large*	**0.81**	[0.60, 0.98]
	*V*_*r*_	**0.80**	[0.58, 0.98]
Panel D			
PostPr	Parameter	Factor Loading	95% Credible Interval
36.7%	*G*_*t*_*_ small*	**0.90**	[0.74, 1.00]
	*G*_*t*_*_ large*	**0.86**	[0.65, 0.99]
	*G*_*r*_*_ small*	**0.91**	[0.74, 0.99]
	*G*_*r*_*_ large*	**0.82**	[0.62, 0.99]
	*V*_*t*_*_small*	−0.34	[−0.99, 0.94]
	*V*_*t*_*_large*	0.71	[−0.33, 0.98]
Panel E			
PostPr	Parameter	Factor Loading	95% Credible Interval
12%	*V*_*t*_*_small*	−0.27	[−0.98, 0.92]
	*V*_*t*_*_large*	0.65	[−0.58, 0.98]
	*a*	0.58	[−0.58, 0.98]
Panel F			
PostPr	Parameter	Factor Loading	95% Credible Interval
0.3%	*G*_*t*_*_ small*	0.89	[0.79, 0.97]
	*G*_*t*_*_ large*	0.78	[0.62, 0.95]
	*G*_*r*_*_ small*	0.83	[0.70, 0.97]
	*G*_*r*_*_ large*	0.68	[0.55, 0.88]
	*a*_*b*_	−0.56	[−0.81, −0.31]
	*b*	−0.47	[−0.76, −0.25]

*V*_*r*_, *a*, *a*_*b*_, and *b* were equal between small and large condition. PostPr = posterior probability that the parameters loaded on the same dominant factor. 95% credible intervals conditional on the specific clustering are presented in brackets. No rotation applied. Loadings are shown in boldface whose 95% credible intervals did not include zero

Summing up, posterior factor analysis of Greene et al.’s (2024) data revealed a clear gist clustering but a weak verbatim clustering. Recollection rejection also displayed a strong association with gist parameters. Guessing processes did not reliably cluster with verbatim or gist parameters.

Taken together, the data of the two papers revealed distinct clustering patterns for verbatim and gist parameters. A major reason could be a point that was mentioned earlier: One paper manipulated phonological gist while the other manipulated semantic gist. In the event, correlations among verbatim parameters were stronger with orthographically related targets and distractors (see Panel B of Table 10) than that with semantically related targets and distractors (see Panel A of Table 10). On the other hand, correlations among gist parameters were stronger with semantically related targets and distractors than with phonologically related targets and distractors (see Panel C of Table 10 and Panel D of Table 10).

**Table 10:** Mean correlations in Greene et al. (2024) and Nieznański and Obidziński (2019) from posterior factor analysis

Panel A: Greene et al. (2024)					Panel B: Nieznański and Obidziński (2019)		
	*V*_*t*_*_small*	*V*_*t*_*_large*	*V*_*r*_			*V*_*t*_	*V*_*r*_
*V*_*t*_*_large*	−0.09				*V*_*r*_	**0.53**	
	[−0.85, 0.80]					[0.17, 0.81]	
*V*_*r*_	−0.39	0.39			*a*	−0.05	0.27
	[−0.91, 0.69]	[−0.40, 0.87]				[−0.27, 0.17]	[−0.05, 0.56]
*a*	−0.08	0.18	0.06				
	[−0.63, 0.61]	[−0.47, 0.71]	[−0.43, 0.49]				
Panel C: Greene et al. (2024)					Panel D: Nieznański and Obidziński (2019)		
	*G*_*t*_*_ small*	*G*_*t*_*_ large*	*G*_*r*_*_ small*	*G*_*r*_*_ large*		*G*_*t*_	*G*_*r*_
*G*_*t*_*_ large*	**0.82**				*G*_*r*_	**0.42**	
	[0.63, 0.94]					[0.05, 0.74]	
*G*_*r*_*_ small*	**0.87**	**0.85**			*b*	0.18	0.13
	[0.73, 0.96]	[0.64, 0.97]				[−0.11, 0.47]	[−0.16, 0.41]
*G*_*r*_*_ large*	**0.73**	**0.72**	**0.79**				
	[0.51, 0.90]	[0.47, 0.90]	[0.58, 0.93]				
*a*_*b*_	**−0.25**	−0.08	−0.11	0.08			
	[−0.47, −0.02]	[−0.34, 0.19]	[−0.39, 0.19]	[−0.24, 0.38]			
*b*	**−0.29**	−0.13	−0.14	0.12			
	[−0.48, −0.07]	[−0.37, 0.13]	[−0.40, 0.14]	[−0.17, 0.41]			

Panel A represents correlations of verbatim parameters and guessing parameter *a* in Greene et al. (2024). Panel B represents correlations of verbatim parameters and guessing parameter *a* in Nieznański and Obidziński (2019). Panel C represents correlations of gist parameters and guessing parameter *b* and *a*_*b*_ in Greene et al. (2024). Panel D represents correlations of gist parameters and guessing parameter *b* in Nieznański and Obidziński (2019). 95% credible intervals are presented in brackets. Mean correlations are shown in boldface whose 95% credible intervals did not include zero. Correlations are on probit scale

Another pattern that merits attention is that the guessing parameter *a* did not load reliably with verbatim or gist parameters, in contrast to the findings at the condition level. Correlations of *V*_*t*_ and *a*, and correlations of *V*_*r*_ and *a* did not credibly differ from zero across all the MCMC samples. Gist parameters were negatively correlated with the guessing parameter *b* and *a*_*b*_ in Greene et al. (2024), but the correlations were modest relative to the correlations at the conditional level (see condition-level correlations in Table 6 and correlations from posterior factor analysis in Table 9).

### Running summary

Factor analysis at the condition level confirmed the independence of the verbatim and gist processes measured by the simplified CR model as gist parameters loaded on one factor, and *V*_*t*_ loaded on a separate factor. The pattern that gist parameters loaded on the same factor corroborated the hypothesis that they index the same memory representations. The condition-level results, however, did not confirm the predicted convergence of verbatim parameters: *V*_*t*_ and *V*_*r*_ loaded on different factors. Considering that recollection rejection is usually difficult to detect with the simplified model, the observed relation between *V*_*t*_ and *V*_*r*_ is subject to the limitation that it might be a restricted range artifact.

To explore that possibility, we conducted posterior factor analysis of the raw data of two studies in which average values of *V*_*r*_ were well above zero. Verbatim processes clustered on the same factor in Nieznański and Obidziński (2019), while gist processes clustered on the same factor in Greene et al. (2024). One study confirmed the notion that verbatim parameters should cluster together, the other study confirmed the notion that gist parameters should cluster together, and both studies confirmed the notion that verbatim and gist parameters should not cluster together. However, the results must be interpretated with caution as reliable estimates of parameter correlations require large numbers of participants and responses per participant.

## Convergent and discriminant validity

To further investigate whether the parameters of the simplified model measure what they are assumed to measure, we reviewed how the model parameters responded to a series of experimental manipulations. These are manipulations that have traditionally been thought to target verbatim or gist memory. Their effects have been observed with a variety of memory tests, including old/new judgment, remember/know judgment, confidence judgment, and so on (e.g., Ahmad et al., 2017; Barber et al., 2008; Brainerd & Reyna, 2023; Fraundorf et al., 2019; McCabe et al., 2009; Montefinese et al., 2015). Verbatim parameters defined by the original CR model have been proved to reacted consistently to a set of manipulations which has been found to effect surface memory, and so have the gist parameters. If the verbatim and gist parameters of the simplified model responded to these manipulations in a similar pattern, we can conclude that the simplified CR model is able to measure what it claims to measure. Extant studies of the simplified CR model have involved six manipulations of this sort: age (older adults vs. younger adults vs. older children vs. younger children), testing delay (short vs. medium vs. long), repetition of study trials (presented once vs. more than once), attention during encoding (full vs. divided), study duration (short vs. medium vs. long) and semantic strength (weak vs. strong gist). The mean values of the six parameters across available experiments for the respective conditions of these manipulations are reported in Table 11.

**Table 11:** Mean values across manipulations, with standard deviations in parentheses

Manipulation		Number of conditions	*V*_*t*_	*V*_*r*_	*G*_*t*_	*G*_*r*_	*a*	*b*
Age	Older adults	20	0.43 (0.24)	0.11 (0.12)	0.55 (0.20)	0.55 (0.27)	0.47 (0.18)	0.23 (0.08)
	Younger adults	20	0.57 (0.22)	0.19 (0.15)	0.53 (0.19)	0.56 (0.23)	0.37 (0.16)	0.22 (0.09)
	Younger adults	6	0.51 (0.37)	0.28 (0.24)	0.60 (0.37)	0.64 (0.33)	0.30 (0.14)	0.09 (0.05)
	Older children	6	0.47 (0.31)	0.13 (0.12)	0.50 (0.39)	0.49 (0.33)	0.41 (0.07)	0.08 (0.04)
	Older children	10	0.79 (0.16)	0.10 (0.13)	0.75 (0.21)	0.73 (0.23)	0.15 (0.10)	0.15 (0.06)
	Younger children	10	0.68 (0.15)	0.20 (0.18)	0.59 (0.21)	0.43 (0.25)	0.48 (0.13)	0.32 (0.13)
Testing delay	Long	2	0.28 (0.13)	0.00 (0.00)	0.32 (0.06)	0.42 (0.11)	0.42 (0.03)	0.48 (0.02)
	Medium	2	0.46 (0.11)	0.01 (0.01)	0.39 (0.13)	0.57 (0.13)	0.33 (0.00)	0.35 (0.04)
	Medium	8	0.44 (0.12)	0.08 (0.10)	0.46 (0.07)	0.60 (0.11)	0.40 (0.05)	0.29 (0.07)
	Short	4	0.73 (0.05)	0.19 (0.17)	0.65 (0.08)	0.81 (0.04)	0.43 (0.06)	0.25 (0.07)
Repetition	Once	6	0.63 (0.31)	0.12 (0.17)	0.38 (0.24)	0.38 (0.28)	0.26 (0.12)	0.29 (0.12)
	Multiple times	6	0.73 (0.23)	0.19 (0.20)	0.50 (0.17)	0.42 (0.29)	0.26 (0.10)	0.28 (0.10)
Attention	Full attention	5	0.58 (0.08)	0.12 (0.11)	0.50 (0.02)	0.69 (0.07)	0.35 (0.04)	0.27 (0.02)
	Divided attention	7	0.47 (0.12)	0.01 (0.02)	0.47 (0.11)	0.60 (0.13)	0.38 (0.04)	0.37 (0.04)
Study duration	Fast	6	0.26 (0.03)	0.01 (0.02)	0.41 (0.12)	0.55 (0.13)	0.48 (0.04)	0.38 (0.11)
	Medium	6	0.46 (0.05)	0.02 (0.02)	0.47 (0.07)	0.66 (0.08)	0.45 (0.08)	0.25 (0.08)
	Slow	6	0.59 (0.03)	0.07 (0.05)	0.52 (0.07)	0.67 (0.03)	0.40 (0.07)	0.23 (0.03)
Semantic strength	Strong	15	0.65 (0.08)	0.17 (0.19)	0.58 (0.17)	0.67 (0.20)	0.31 (0.11)	0.22 (0.12)
	Weak	15	0.64 (0.12)	0.12 (0.13)	0.30 (0.17)	0.22 (0.17)	0.26 (0.09)	0.22 (0.12)

For age, older adults were >59 years old; younger adults were 18–35 years old; older children were 8–10 years old; younger children were 4–5. For testing delay, long delay was >12 hours; medium delay was 1–5 min; short delay was 2–30 s. For repetition, the multiple times condition refers to the situation where materials were presented two or four times. For study duration, fast vs. medium vs. slow presentation was 0.75 s vs. 1.5 s vs. 4 s, 1 s vs. 2 s vs. 4 s, and 2 s vs. 4 s vs. 6 s

The first four manipulations have affected verbatim and gist parameters differentially in prior studies using the original CR model. To begin, a developmental trend has been identified between early childhood and young adulthood, in which verbatim and gist parameters both exhibit substantial improvement (for a review, see Brainerd & Reyna, 2023). Further, comparisons between younger and older adults have revealed that verbatim parameters decline more than gist parameters (Brainerd et al., 2014; Brainerd & Reyna, 2015). Second, both verbatim and gist parameters decrease over long testing delays, with the decline in verbatim parameters being more pronounced (Brainerd et al., 2001, 2003, 2006; Mojardín et al., 2003, 2005; Pansky, 2012; Reyna & Kiernan, 1994, 1995). Note that the forgetting rates of verbatim and gist memory can be moderated by specific encoding instructions and cues (Hu & Yang, 2023; Hu et al., 2021). Third, repetition increases both verbatim and gist parameters, with the increases being greater for verbatim parameters (Brainerd et al., 1999, 2001). The gist effect peaks after fewer repetitions than the verbatim effect (Brainerd & Reyna, 2005). Therefore, repetition should increase both the verbatim and gist parameters of simplified CR, with the verbatim effect being larger. Next, divided attention strongly suppresses verbatim parameters, relative to full attention (Odegard & Lampinen, 2005). With respect to the study duration manipulation, it elevates both verbatim and gist parameters, with gist increases occurring at shorter durations than verbatim increases (Brainerd & Reyna, 2005; Leding & Lampinen, 2009). Finally, concerning semantic strength, multiple manipulations that strengthen the semantic connections between list items have been investigated with the original CR model (e.g., Brainerd et al., 2001, 2011, 2020). Such manipulations have affected gist parameters without affecting verbatim parameters. Hence, the expectation is that the simplified model would exhibit the same pattern.

Summing up, prior studies with the original CR model establish some baseline patterns that can be used to investigate the convergent and discriminant validity of the simplified model. Convergent validity could be demonstrated by showing that (a) its verbatim and gist parameters increase between early childhood and young adulthood while its verbatim parameters decrease thereafter; (b) verbatim parameters and gist parameters decline over testing delays; (c) repetition improves both verbatim and gist parameters; and (d) verbatim and gist parameters both increase as study duration increases. Discriminant validity could be established by showing that (a) verbatim parameters decline more than gist parameters between early and late adulthood; (b) verbatim parameters decline more than gist parameters over testing delays; (c) repetition increases verbatim parameters more than gist parameters; (d) divided attention suppresses verbatim parameters more than gist parameters; (e) verbatim parameters increase more than gist parameters as study duration increases; and (f) semantic activation increases gist parameters more than verbatim parameters.

Findings on these convergent and divergent validity possibilities are reported in the sections that follow. For each manipulation, we review the effects that have been observed in individual studies. During the course of this review, consistent patterns of parametric effects will become apparent for some manipulations.

### Age

Four age groups were examined across these studies: older adults (>59), younger adults (18–35), older children (8–10) and younger children (4–5). Six studies compared older adults versus younger adults; one compared older children versus younger adults; and one compared older children (8–10) versus younger children (4–5). The participant samples of the remaining 18 articles were confined to younger adults.

Inspection of the results of the six studies that compared younger versus older adults reveals age-related declines in the two verbatim parameters (*V*_*t*_ and *V*_*r*_) but not in the two gist parameters (*G*_*t*_ and* G*_*r*_). This pattern is theoretically predicted (see Greene & Naveh-Benjamin, 2023b), and it was preserved across variations in semantic activation (Abadie et al., 2021; Abadie & Guette, 2025) and emotional valence (Xiao et al., 2015). Further, these differences appeared for both short- and long-term memory tasks (Greene & Naveh-Benjamin, 2020, 2022a) and for different presentation durations (Greene & Naveh-Benjamin, 2024). Moreover, there were discrepancies between the age-related declines in *V*_*t*_ and *V*_*r*_ in some instances. There are studies in which *V*_*t*_ decreased with age, but *V*_*r*_ did not show reliable trends, with both strongly associated words (Abadie & Guette, 2025) and weakly associated words (Abadie et al., 2021; Abadie & Guette, 2025) and with words that differed in emotional valence (Xiao et al., 2015). However, in some cases, *V*_*r*_ was invariant between two age groups because it was near-zero in both age groups and was fixed at zero to improve fit (Greene & Naveh-Benjamin, 2024). Finally, aging effects were different with short-term and long-term memory tasks. In a study examining associative memory for pictures of human faces and scenes, *V*_*t*_ was comparable between two age groups while *V*_*r*_ declined with age on a short-term memory test, whereas on a long-term memory test, only *V*_*t*_ showed reliable age declines (Greene & Naveh-Benjamin, 2022a).

There was also evidence that the verbatim and gist parameters both increased during early development. For younger adults versus children, estimates of both verbatim parameters and both gist parameters were larger in younger adults on both short-term and long-term memory tests (Abadie & Rousselle, 2023; Rousselle, 2023).

### Testing delay

Across studies, three types of test delays were implemented:

- Tests administered <30 s after study;
- Tests administered 30 s–5 min after study;
- Tests administered ≥12 hours after study.

Some studies included two testing delays—for example, short vs. medium delay (Greene & Naveh-Benjamin, 2022a, b), short vs. long-delay (Chapter 5 Experiment 2 of Rousselle, 2023), medium vs. long delay (Greene & Naveh-Benjamin, 2023b). Others included only one—for example, short delay (Abadie & Rousselle, 2023; Chapter 5 Experiment 1 and Chapter 7 of Rousselle, 2023), medium delay (Nieznański et al., 2024; Chapter 6 of Rousselle, 2023); long delay (Macera & Daurat, 2018; Experiments 1 and 2 in Stahl & Klauer, 2008).

For short versus medium delay, *V*_*t*_ declined remarkably in both younger and older adults, but *V*_*r*_ did not differ between the two age groups (Greene & Naveh-Benjamin, 2022a). The two gist parameters (*G*_*t*_ and* G*_*r*_) also decreased from short to medium delay in both younger and older groups (Greene & Naveh-Benjamin, 2022a). In a study comparing an encoding condition with full attention to a divided attention condition, *V*_*t*_ decreased from short to medium delay in both full and divided attention conditions, but *V*_*r*_ was similar between the two conditions (Greene & Naveh-Benjamin, 2022b). Change in the two gist parameters was also moderated by the level of attention at encoding. *G*_*t*_ and *G*_*r*_ diminished from short to medium delay in the divided attention condition, whereas *G*_*t*_ remained constant after delay in the full attention condition (Greene & Naveh-Benjamin, 2022b). Taken together, from short-term memory to immediate long-term memory, *V*_*r*_ did not decrease as much as *V*_*t*_, *G*_*t*_, and *G*_*r*_.

Finally, contrasts between medium and long delays revealed sizeable reductions in *V*_*t*_ but not in the remaining parameters, except that when list words were presented twice, *G*_*r*_ declined after 24 hours (Greene & Naveh-Benjamin, 2023b).

### Repetition

Three studies examined the effects of repetition. Repeated presentation greatly increased verbatim memory for old items (*V*_*t*_) in all cases (Abadie & Guette, 2025; Greene & Naveh-Benjamin, 2023b; Stahl & Klauer, 2008). It also elevated recollection rejection of similar distractors (*V*_*r*_) in older adults (Abadie & Guette, 2025) and the gist parameter for similar distractors (*G*_*r*_) (Greene & Naveh-Benjamin, 2023b).

### Attention

The effects of divided attention were examined in three studies. Concurrent pitch tasks thar required discrimination of three pitches during the study phase significantly decreased verbatim memory for old items and gist memory for similar distractors (*V*_*t*_ and *G*_*r*_) but not recollection rejection of distractors or gist memory for old items (*V*_*r*_ and *G*_*t*_) (Greene & Naveh-Benjamin, 2022c, 2023a). Another study revealed that this manipulation strongly suppressed *V*_*r*_ in working memory tasks, while suppressing *V*_*t*_, *G*_*t*_ and *G*_*r*_ in long-term memory tasks (Greene & Naveh-Benjamin, 2022b). These authors also investigated two easier divided attention manipulations that involved pitch discrimination (Greene & Naveh-Benjamin, 2023a). The easier of the two, which required responses to a single pitch, failed to affect either verbatim or gist memory. The harder one, which required discrimination between two pitches, suppressed *V*_*t*_ only. The overall picture for divided attention, then, is that it consistently affects verbatim traces’ ability to support correct acceptance of old items.

### Study duration

The impact of study duration on the parameters of the simplified model was evaluated in two studies. The target items were displayed at a fast, medium or slow rate (0.75s vs. 1.5s vs. 4s, 1s vs. 2s vs. 4s, or 2s vs. 4s vs. 6s) during the study phase (Greene & Naveh-Benjamin, 2023c, 2024). When the study durations were manipulated between-participant, *V*_*t*_ increased reliably across the three exposure durations, but the verbatim parameter for similar distractors was not affected. The two gist parameters increased reliably from the short to the long duration but not from the medium to the long duration, and *G*_*r*_ also increased from the short to the medium duration (Experiment 1 in Greene & Naveh-Benjamin, 2023c). Expanding the duration of intervening tasks in the short and medium durations to match the total length of the study phase across conditions produced the same results, except that *V*_*t*_ was not elevated from the short to the medium duration and *G*_*r*_ increased across the three durations (Experiment 2 in Greene & Naveh-Benjamin, 2023c). Study duration had somewhat different effects in younger versus older adults when the durations were manipulated within-subject. With younger adults, *V*_*t*_ increased progressively with longer durations, *G*_*r*_ only increased from the short to the medium duration while *G*_*t*_ was not affected. With older adults, increases in *V*_*t*_ were detected from the short to the medium and from the short to the long duration. In the meantime, *G*_*t*_ improved from the medium to the long duration, and *G*_*r*_ improved from the short to the medium duration only (Green & Naveh-Benjamin, 2024).

### Semantic strength

Semantic strength was manipulated by presenting one exemplar versus multiple exemplars of taxonomic categories or by presenting semantically related word lists versus unrelated word lists. A consistent finding of these studies is that increasing semantic strength increased the values of both gist parameters (*G*_*t*_ and *G*_*r*_; Abadie et al., 2021; Abadie & Guette, 2025; Gong et al., 2016; Rousselle, 2023; Stahl & Klauer, 2008). In contrast, its effect on verbatim memory varied across these studies. In experiments with longer retention intervals between study and test for word lists (5 min in Abadie et al., 2021; Abadie & Guette, 2025; and 24 hours in Stahl & Klauer, 2008), *V*_*t*_ and *V*_*r*_ were not affected by semantic strength. In a similar study with pictures, increasing the number of exemplars of each category reduced both *V*_*t*_ and *V*_*r*_, indicating that high levels of gist activation hinder correct identification of old items and rejection of similar distractors through retrieval of verbatim traces (Gong et al., 2016).

### Running summary

The convergent and discriminate validity of the simplified CR model were confirmed by various findings of these studies. Beginning with convergent validity, verbatim parameters responded to manipulations that are assumed, theoretically and based on prior findings, to influence verbatim memory—specifically, age, testing delay, repetition, attention, and study duration. From early childhood to early adulthood, *V*_*t*_ and *V*_*r*_ increased substantially and then declined substantially between early and late adulthood. As the delay between study and test increased, *V*_*t*_ declined significantly whereas *V*_*r*_ did not display a consistent pattern. When study items were presented more than once, *V*_*t*_ and *V*_*r*_ both increased. Divided attention tasks during study consistently decreased *V*_*t*_ and sometimes decrease *V*_*r*_ , depending on factors such as testing delay and level of difficulty of divided attention tasks. Finally, extending study duration consistently boosted *V*_*t*_.

Continuing with convergent validity, the gist parameters were also sensitive to manipulations that, theoretically and empirically, ought to affect gist memory—specifically, age, testing delay, repetition, attention, study duration and gist activation. *G*_*t*_ and* G*_*r*_ increased steadily from early childhood to early adulthood and remained invariant thereafter. They also decreased from short to medium delays. *G*_*r*_ was elevated by repetition and was suppressed by divided attention tasks, but only by the most difficult of such tasks. The two gist parameters increased dramatically from short to long study durations. Lastly, both parameters were consistently elevated by gist activation manipulations.

Turning to discriminate validity, verbatim and gist parameters behaved differently for some manipulations that are assumed to selectively affect either verbatim or gist memory—specifically, aging, long testing delays, repetition, study duration, and gist activation. First, healthy aging is assumed to produce declines in verbatim memory while gist memory is largely spared (for a review, see Greene & Naveh-Benjamin, 2022a). Consistent with that, the declines in the verbatim parameters between earlier and later adulthood were much larger than the declines in gist parameters. After long delays, *V*_*t*_ decreased notably whereas the two gist parameters were more stable. As also expected, repetition increased *V*_*t*_ much more than it increased the gist parameters. The last discriminant validity result pertains to the effects of study durations; that gist effects ought to occur earlier than verbatim effects. Due to limited studies tackling this possibility, we did not obtain a conclusive pattern. In most cases, *V*_*t*_ increased across the short, medium and long study durations, but gist parameters were more variable. In a within-participant design, *G*_*r*_ increased from the short to the medium duration in both younger and older adults, whereas *G*_*t*_ in older adults was boosted only from the medium to the long duration. In a between-participant design, *G*_*t*_ was not affected by the medium duration compared to the short duration while *G*_*r*_ was elevated consistently across the three duration levels. Note that although gist and verbatim parameters were not completely dissociative in certain situations, the two still accounted for unique variance. As illustrated in the factor analyses, correlations between verbatim and gist parameters were very low, far below the discriminant validity criterion.

## Discussion

The goal of the present study was to review findings that bear on the validity of the simplified CR model. Exploratory factor analyses were conducted to determine whether the structure of its parameter space conformed to theoretical expectations, which it did for gist parameters but not for verbatim parameters. Theoretically, verbatim and gist parameters should load on different factors, gist parameters should load on one while verbatim parameters load on another. There was a verbatim factor on which one of the bias parameters and the verbatim parameter for old items both loaded strongly, in opposite directions. However, the verbatim parameter for similar distractors did not load on that factor and, instead, loaded by itself on a third factor. Then, we conducted posterior factor analysis on data from two studies. One provided clear verbatim clustering while the other one proved clear gist clustering. Thus, the two verbatim parameters loaded on the same factor with specific experimental procedures.

Against that background, we conclude by briefly discussing three remaining conceptual issues. Those issues are the model’s parameter space, the model’s inability to measure phantom recollection, and an alternative paradigm, repeated-measures CR, which implements the same design as original CR except for varying recognition instructions (V, G, VG) within-participant.

### Structure of the parameter space

The factor analysis at the condition level produced a violation of fuzzy-trace theory’s assumption that *V*_*t*_ and *V*_*r*_ should load on a common factor because both measure retrieval of verbatim traces of old items. Nevertheless, the fact that *V*_*t*_ and *V*_*r*_ occupied different factors does not imply a fundamental flaw in the simplified CR model. To begin with, analysis of one study that produced moderate values of *V*_*r*_ (Nieznański & Obidziński, 2019) yielded definite verbatim clustering, indicating that *V*_*t*_ and *V*_*r*_ conformed to theoretical prediction. Moreover, *V*_*r*_ was sensitive to a classic verbatim retrieval manipulation, namely, testing similar distractors immediately after testing their corresponding targets (Stahl & Klauer, 2008). *V*_*r*_ was significantly larger compared to a condition in which related distractors were tested before the corresponding targets (.72, vs. .05, and .73 vs. .33 in Experiment 5 and 6 of Stahl & Klauer, 2008). To conclude, *V*_*t*_ and *V*_*r*_ was able to measure verbatim memory elicited by targets and related distractors, respectively, aligning with the model’s assumption. Weak association of *V*_*t*_ and *V*_*r*_ across studies relates to the difficulty of verbatim retrieval elicited by related distractors on forced-choice tasks.

A possible explanation for the weak association of *V*_*t*_ and *V*_*r*_ is that the simplified paradigm makes recollection rejection much more difficult than the original paradigm, reducing covariance with *V*_*t*_. That is consistent with the aforementioned fact that the mean value of *V*_*r*_ across studies was only .12 (*SD* = .15), with 27% of the parameter estimates equal to zero. After re-fitting the raw data with the 6-parameter model, the mean value of reestimated *V*_*r*_ was only 0.10 (*SD* = 0.15), with 44% of them equal to zero. As a comparison, among 609 conditions in the original CR corpus, only 52 (0.09%) produced zero values of recollection rejection (Brainerd et al., 2022). Although the magnitude of recollection rejection measured by the two models cannot be directly contrasted, high proportion of zero values of recollection rejection in the simplified CR corpus indicated that the simplified procedures produced more low values of recollection rejection. Considering that the data corpus for the original CR model is much larger than the corpus for the simplified model, the latter may include a disproportional number of low values of *V*_*r*_. Therefore, we assessed recollection rejection estimated from similar conditions to ensure valid comparisons. First, we compared recollection rejection estimated from conditions where pictures were used as stimuli and no long delay was implemented. Pictures, as compared with words, elicit stronger verbatim memories. In the original CR corpus, 17 conditions involved picture stimuli, while in the simplified CR corpus, 58 conditions used pictures, among which 44 employed face–scene pairs and 16 employed individual images. The simplified CR corpus contains more zero values (36%) than the original CR dataset (29%). It appears that recollection rejection elicited by picture stimulus is easier with the standard CR procedure than with the multiple-choice procedure.

Next, we compared recollection rejection estimated from the Deese, Roediger, and McDermott (DRM) lists (Deese, 1959; Roediger & McDermott, 1995), or DRM-like lists. DRM lists were constructed such that words on the same list are closely associated with an unpresented critical lure which, at test, is able to provoke vivid illusory memory of its prior presentation. We selected 410 and 93 conditions using DRM or DRM-like lists and without long delays in the original CR and simplified CR corpora respectively. More zero values appeared with the simplified CR procedures (31%) than with the original CR procedures (12%). Taken together, the simplified CR paradigm produced more zero values of recollection rejection from both word lists and pictures relative to the original CR paradigm. It is therefore reasonable to infer that recollection rejection is more difficult with forced-choice tests.

The remaining questions is why, if so, recollection rejection for RDs is more difficult with the simplified procedure? One hypothesis pertains to the test instructions. The original CR employs three types of instructions (V?, G?, VG?), which require yes/no responses. Previous studies have found that accuracy was higher with new words accompanied by Old? question (*No* responses) than new words accompanied by New? questions (*Yes* responses; Brainerd et al., 2022). Accuracy was also higher with old words accompanied by New? questions (*No* responses) than old words accompanied by Old? questions (*Yes* responses). In other words, subjects were better at rejecting the quired state of a given test probe. Consequently, the V? instructions for RDs facilitate retrieval of verbatim information to a greater extent as compared to three choices (old, new similar, and new novel). Verbatim retrieval induced by targets may also be affected by the lack of detailed instructions in simplified CR.

**Fig. 2:**
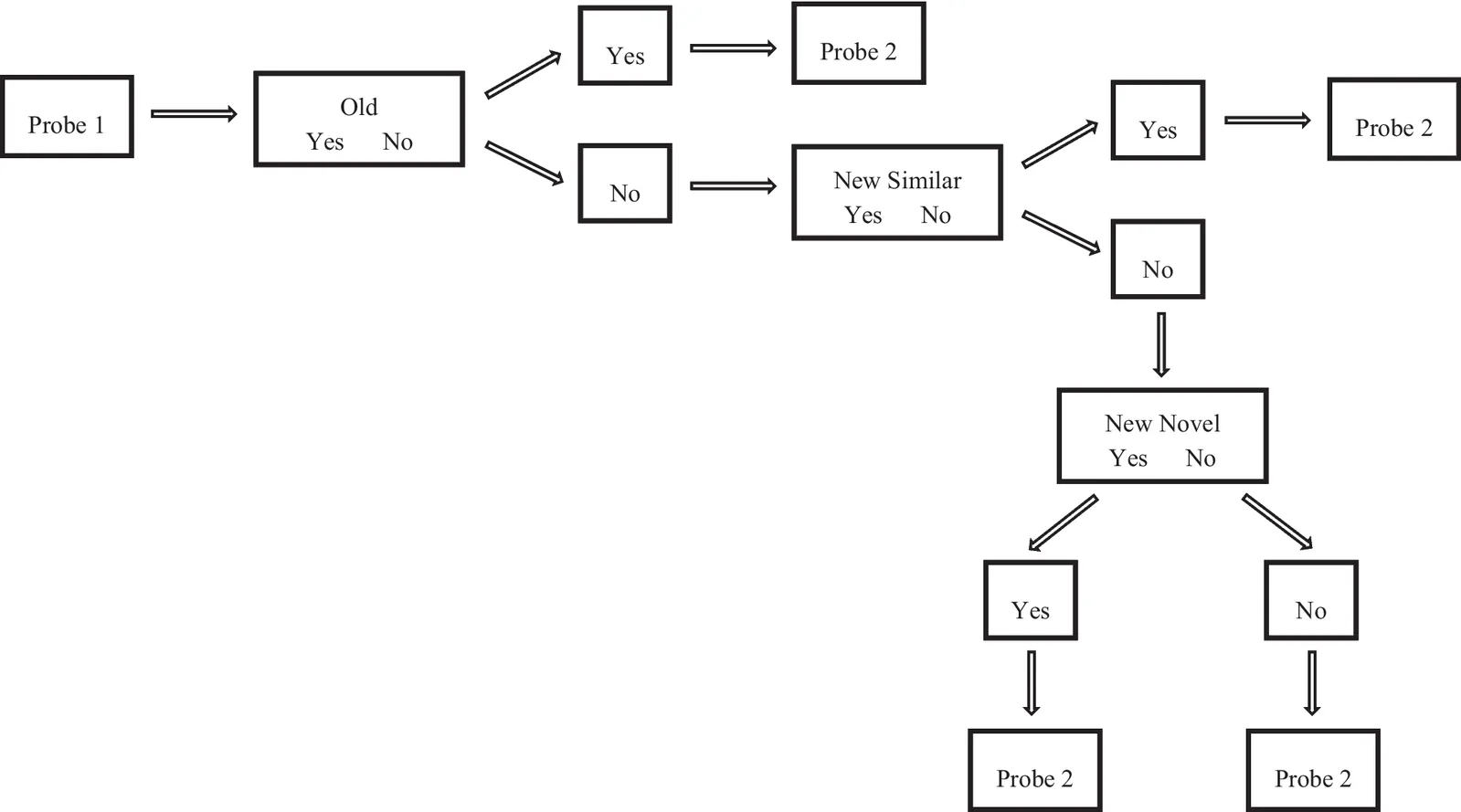
Procedures of sequential presentation

Finally, we propose an experiment using a sequential presentation of the three options to examine whether the specific V instruction affects recollection rejection relative to the forced-choice format. As illustrated in Fig. 2, each test probe follows a three-step sequence. The *old* option is presented first, with *yes* and *no* choices. If *yes* is selected, the test moves to the next probe and if *no* is selected, the *new similar* option appears. A *yes* response then advances to the next probe, again starting with the *old* option. If *no* is selected, the third option, *new novel*, is presented with yes/no buttons. Data with the same structure can be obtained from the sequential presentation procedures (*p*(T)_T_, *p*(RD)_T_,* p*(UD)_T_,* p*(T)_RD_,* p*(RD)_RD_,* p*(UD)_RD_,* p*(T)_UD_,* p*(RD)_UD_, and* p*(UD)_UD_) and the same model (Eqs. 10–18) can be used to estimate the parameters. Therefore, the values of *V*_*r*_ obtained from the sequential presentation and simultaneous presentation can be contrasted. If the sequential presentation yields a larger value, the hypothesis is confirmed that simultaneous presentation impairs recollection rejection, and the decline in *V*_*r*_ could be alleviated by sequential presentation.

### Phantom recollection

The original CR model introduced a phantom recollection parameter to measure the phenomenon of illusory vivid recollection of prior “presentation” of similar distractors. This process is of critical importance in the false-memory literature because tasks that generate the highest levels of false memory usually generate high levels of phantom recollection (Arndt, 2012; Brainerd & Reyna, 2005; Singer & Spear, 2015). In the Deese/Roediger/McDermott (DRM; Deese, 1959; Roediger & McDermott, 1995) illusion, for instance, participants may express phantom recollection of similar distractors as often as they express true recollection of old items (Arndt, 2012). With the simplified model, there is no room for such a parameter because there are already six parameters and only 6 degrees of freedom. However, there would be room if the parameter space were reduced from six to five. One approach is to set *V*_*r*_ to zero (Stahl & Klauer, 2009). Another approach is to expand the experimental design to supply other judgments (e.g., adding a remember/know judgment after subjects choose “target” for a test probe; Gong et al., 2016).

In studies that implemented the *V*_*r*_ = 0 approach, phantom recollection has reacted to manipulations that affect the gist parameters, which is predicted theoretically (see Brainerd et al., 2001). For instance, younger adults had higher phantom recollection values than children, echoing the known developmental trend for gist memory (Abadie & Rousselle, 2023). Also, increasing the number of same-category exemplars in a categorized list boosted phantom recollection (Gong et al., 2016; Stahl & Klauer, 2009), as theory predicts.

### Repeated-measures CR

The incentive for developing the simplified CR paradigm was to reduce the sample sizes that are required for sufficient power to detect effects with the model’s parameters, thereby increases the efficiency of CR experiments. That was accomplished by switching to a forced-choice procedure and presenting the same test instruction with all the test probes and across all the subjects. However, that modification reduced the degrees of freedom that were available for modeling from 9 to 6.

In closing, it is important to note that efficiency goal can also be achieved without sacrificing any of the original CR model’s degrees of freedom by varying the three CR conditions within subjects rather than between subjects. To date, this repeated-measure CR procedure has been implemented in a modest number of studies to examine such things as disjunction fallacies in episodic memory (Brainerd et al., 2010) and the memory effects of latent semantic attributes (Brainerd et al., 2023). It has also been used to measure the speed of verbatim and gist access during retrieval (Brainerd et al., 2019).

Relative to simplified CR, repeated-measures CR has the key advantage, as we said, that it generates the same number of free empirical probabilities as the original CR paradigm, and hence, all the parameters of the original model (Table 1) can be estimated. In principle, then, if the goal is only to increase experimental efficiency, the repeated-measures CR is an attractive alternative to simplified CR. However, repeated-measures CR is not without deficits. For instance, participants are likely to adopt different strategies on the three types of memory probes, which is a new source of error variance that can comprise fit (see also in Stahl & Klauer, 2008).

## Concluding comments

In conclusion, the data argue that the simplified CR model is a valid and reliable technology for extracting measures of verbatim retrieval for old items and gist retrieval for both old and similar items in recognition. Whether it is a valid and reliable method of extracting measures of verbatim retrieval for similar distractors remains an open question. Available findings for some classic verbatim and gist manipulations supply solid evidence of convergent and discriminant validity. There, verbatim and gist parameters responded to manipulations that are thought to affect verbatim and gist retrieval, respectively.

The simplified paradigm has important strengths, but the data indicate that it has two notable limitations, relative to the original procedure. First, as just noted, it remains unclear what the *V*_*r*_ parameter measures, and estimates of this parameter are so low that, psychometrically, it is inherently difficult to detect effects with it. Second, the procedure is unable to measure the theoretically important process of phantom recollection, which has stimulated considerable theoretical attention because it is an enduring feature of paradigms that generate the highest levels of false memory.

## Data Availability

All data are available at https://osf.io/f739y/?view_only=be6552a4d0bf47e482d853ad8beac9a3.

## Footnotes

1 A confirmatory factor analysis was also conduced to assess whether the parameter space of the simplified CR paradigm was composed of a verbatim dimension and a gist dimension. However, the two-factor model produced a poor fit to the data, indicating that the simplified CR parameters did not load on two orthogonal factors as expected.

2 Rotation method (e.g., varimax and promax) did not change the pattern described below. For illustrative simplicity, we reported results obtained without rotation. The same logic applied to the next section.

## Code availability

Analysis scripts are available at https://osf.io/f739y/?view_only=be6552a4d0bf47e482d853ad8beac9a3.
